# Rethinking Cardiopulmonary Bypass Management in The Digital Health Era

**DOI:** 10.1016/j.mcpdig.2026.100343

**Published:** 2026-02-01

**Authors:** Youssef El Dsouki, Ignazio Condello, Roberto Lorusso

**Affiliations:** a Department of Health, Medicine and Life Sciences, Cardiovascular Research Institute Maastricht (CARIM), Maastricht University, The Netherlands; b Department of Cardio-Thoracic Surgery, Maastricht University Medical Centre (MUMC), The Netherlands; c School of Medicine and Surgery, University of Varese Insubria, University of Insubria, Italy

## Abstract

Minimally invasive and robotic cardiac surgery have been developed to reduce surgical trauma, shorten recovery, and improve cosmetic and functional outcomes. However, these approaches often require longer cardiopulmonary bypass (CPB) and aortic cross-clamp times than conventional full sternotomy, and CPB duration remains an independent predictor of postoperative morbidity and mortality, particularly in frail patients with reduced physiological reserve. The resulting less invasive access/prolonged extracorporeal support duration paradox poses a major physiological and clinical challenge. Contemporary evidence from randomized and observational studies reports that while minimally invasive and robotic procedures achieve comparable or improved survival and functional recovery, extended CPB and aortic clamp times can amplify the risk of renal dysfunction, neurological events, and systemic inflammation. Advances in digital health are now transforming intraoperative perfusion management: high-frequency data acquisition, automated oxygen delivery and consumption analytics, and real-time artificial intelligence-driven predictive models enable early detection of perfusion imbalance and metabolic distress. Integration of these data streams within interoperable platforms and patient-specific digital twins may allow dynamic modeling of perfusion adequacy and adaptive control of pump flow, temperature, and hemodynamics. By converting CPB duration from a static procedural metric into a digitally monitored, optimizable variable, precision perfusion could reconcile minimal invasiveness with physiological safety. Future research should validate these digital frameworks in multicenter studies and establish standards for transparency, interoperability, and ethical implementation in real-world cardiac surgery.

**Article Highlights**

• Prolonged cardiopulmonary bypass remains a major determinant of postoperative morbidity, particularly in frail patients undergoing minimally invasive cardiac surgery.
• Conventional perfusion monitoring relies on global systemic surrogates and lacks reliable real-time assessment of regional organ perfusion.
• Digital perfusion platforms enable continuous data acquisition and predictive modeling of physiological stress during cardiopulmonary bypass.
• Machine learning and digital twin concepts offer a framework for anticipatory, patient-specific perfusion management.
• Precision perfusion may attenuate time-dependent risk and extend the duration of low-risk cardiopulmonary bypass.

Over the past 2 decades, cardiac surgery has evolved through a continuous process of technological refinement aimed at minimizing surgical trauma and improving patient recovery and ultimate outcome.^1^^,^^2^ The emergence of minimally invasive and robotic techniques has transformed the operative landscape, particularly for mitral, aortic, and coronary procedures.^3^^,^^4^^,^^5^ By reducing incision size and avoiding full median sternotomy, these approaches have reported clear benefits in terms of postoperative pain, transfusion requirements, length of stay, and cosmesis, without compromising the surgical results.^4^^,^^6^ For many patients, this paradigm shift of surgical approach has translated into faster functional recovery and improved quality of life. However, this advancement has also introduced a physiological and procedural paradox: smaller incisions and reduced trauma come at the cost of longer cardiopulmonary bypass (CPB) and aortic cross-clamp durations. Compared with conventional sternotomy, minimally invasive and robotic procedures typically require additional perfusion and ischemic times due to limited exposure, restricted instrument motion, and more complex cannulation and deairing strategies, with almost inevitable longer operative times and related exposure.^7^, ^8^, ^9^, ^10^ These temporal extensions, sometimes exceeding 30 minutes beyond standard CPB durations, may appear modest from a technical perspective, yet they represent a clinically significant burden, especially for patients with limited physiological reserve.^9^^,^^11^ A consistent body of evidence has reported that CPB and cross-clamp times are among the most powerful independent predictors of postoperative morbidity and mortality across all cardiac surgical procedures.^12^^,^^13^ Longer bypass duration correlates directly with the incidence of acute kidney injury, low cardiac output syndrome, neurological dysfunction, and systemic inflammatory response.^14^^,^^15^ Importantly, the detrimental impact of time is nonlinear and magnified in frail or elderly individuals, where microcirculatory reserve, metabolic flexibility, and inflammatory control are inherently compromised.^15^^,^^16^^,^^17^ In such patients, each additional minute of bypass carries incremental risk.^5^^,^^18^ This time-dependent vulnerability exposes a fundamental limitation of current intraoperative management: the inability to dynamically adapt perfusion parameters to real-time physiological reaction.^19^^,^^20^ Traditional perfusion strategies, despite continuous refinement in oxygenator and pump design as well as flow algorithms, still rely heavily on operator experience and intermittent measurements. In this review, we summarize current evidence and propose a digital framework to mitigate time-dependent risk during CPB.

## Themes

### The Time Paradox in Minimally Invasive and Robotic Cardiac Surgery

The development of minimally invasive and robotic cardiac surgery has marked a major milestone in the evolution of cardiovascular care. Numerous studies have confirmed that limited-access approaches via right minithoracotomy, trans-axillary, hemisternotomy, or totally endoscopic ports, yield equivalent or improved clinical outcomes compared with full sternotomy.^1^, ^2^, ^3^^,^^5^^,^^18^, ^19^, ^20^ In the randomized multicenter trial by Akowuah et al,^1^ right minithoracotomy for mitral valve repair achieved similar mortality and valve competence but resulted in shorter recovery, reduced transfusion needs, and higher patient satisfaction. Meta-analyses by Williams et al^2^ and Eqbal et al^3^ corroborated these findings, reporting lower infection rates and hospital stay, albeit at the cost of considerably longer CPB and aortic cross-clamp times. This paradox reflects a shift from mechanical simplicity to technological complexity. The introduction of robotic systems and video-assisted instrumentation requires additional setup, docking, and perfusion steps, often extending CPB and cross-clamp time duration by 20-30 minutes or more.^4^^,^^6^, ^7^, ^8^, ^9^, ^10^ Historically, these time penalties were accepted as the price of minimal invasiveness. The concept of time as a biological variable has gained attention, recognizing that the same procedural delay can have vastly different metabolic consequences depending on patient reserve, perfusion strategy, and real-time adaptation.^21^^,^^22^ Before the digital era, perfusion data were collected intermittently and documented manually, making it nearly impossible to correlate minute-to-minute physiological changes with outcomes. This fragmented data acquisition limited the understanding of how prolonged CPB impacts organ function. The current transition toward digital perfusion and continuous data capture allows unprecedented granularity, transforming isolated parameters into longitudinal and precision-based biological change monitoring during CPB that can guide intraoperative strategy and related management.

### Duration of CPB as a Physiological Stressor

**Figure 1:**
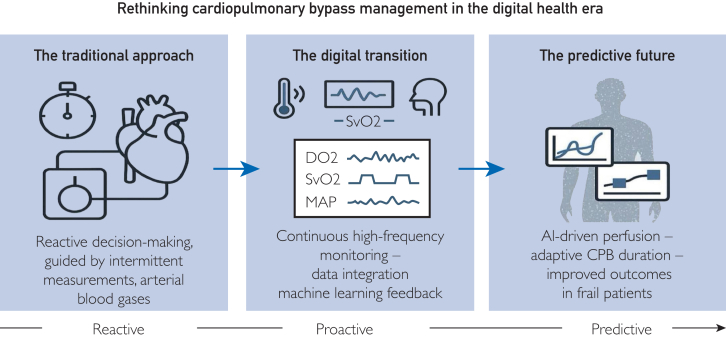
Time-dependent accumulation of organ injury during cardiopulmonary bypass. This conceptual figure illustrates the progressive and nonlinear accumulation of organ-specific and systemic injury with increasing cardiopulmonary bypass duration, as consistently reported across observational studies and meta-analyses. Prolonged bypass exposure is associated with a cumulative rise in renal, cardiac, neurological, inflammatory, metabolic, and peripheral ischemic complications, ultimately leading to multiorgan dysfunction and mortality. The figure emphasizes cardiopulmonary bypass time as a cumulative physiological stressor rather than a purely procedural variable.

The adverse effects of prolonged CPB have been recognized since the early days of open-heart surgery. Salis et al^12^ reported a stepwise increase in morbidity and mortality with every additional half hour of bypass time, independent of patient risk or procedure type. Doenst et al^14^ extended this observation, showing that the relationship between aortic cross-clamp duration and mortality is not linear but exponential beyond a threshold of ∼90 minutes, beyond which each additional minute confers a disproportionately higher risk (Figure 1). Al-Sarraf et al^13^ further confirmed that even after adjustment for surgical complexity, perfusion time remains an independent determinant of renal and respiratory complications. These findings underscore that CPB duration acts not only as a measure of technical complexity but also as a physiologic stress index.^11^, ^12^, ^13^, ^14^, ^15^, ^16^, ^17^ In the predigital perfusion era, the capacity to detect and respond to these dynamic pathophysiologic changes was limited. Perfusionists relied on static snapshots arterial pressure, venous saturation, and occasional lactate measurements often interpreted with considerable delay. The lack of data consistency across devices compounded this limitation; different consoles recorded variables in incompatible formats, preventing real-time trend analysis. Traditional perfusion monitoring during CPB still relies predominantly on global systemic surrogates, such as oxygen delivery (DO_2_), mixed venous oxygen saturation (SvO_2_), arterial pressure, and intermittent lactate measurements. While these parameters are essential, they provide only an indirect and delayed representation of tissue perfusion and offer little insight into regional or organ-specific oxygenation.^23^^,^^24^ A satisfactory SvO_2_ does not exclude selective hypoperfusion, and lactate elevation often reflects metabolic distress that occurred earlier during bypass rather than ongoing ischemia. Experimental and clinical evidence reports that organ injury during CPB may develop despite apparently adequate systemic indices. In particular, renal medullary hypoxia can occur without detectable changes in SvO_2_ or lactate levels, highlighting the vulnerability of the kidney to prolonged bypass even under acceptable perfusion conditions. Similarly, peripheral limb ischemia may remain clinically silent until reperfusion, when washout of potassium, lactate, and other metabolites unmasks the injury. These observations underscore that, despite technological advances, truly reliable real-time measures of regional organ perfusion are still lacking. Current digital and predictive perfusion tools should therefore be interpreted as probabilistic risk estimators rather than direct measurements of organ perfusion, and claims of universally safer CPB management must be tempered accordingly.

### Frailty and Reduced Physiological Reserve

Frailty represents the biological substrate through which time exerts its most detrimental effects. It is a multidimensional construct encompassing sarcopenia, inflammation, microvascular dysfunction, and metabolic rigidity.^15^^,^^16^^,^^21^ Traditional frailty scores are static and largely preoperative, capturing demographic characteristics and comorbidity-related risk but failing to reflect intraoperative physiological resilience. The concept of digital frailty assessment refers to an emerging, nonstandardized approach that integrates continuous intraoperative physiological signals (such as oxygen delivery trajectories, SvO_2_ dynamics, pressure-flow relationships, and metabolic markers), perioperative clinical data, and, when available, imaging or functional biomarkers.^25^ Rather than generating a single score, digital frailty aims to characterize patient-specific tolerance to physiological stress in real-time.^23^^,^^26^ This data-driven phenotyping enables dynamic estimation of metabolic exhaustion and reduced compensatory reserve during prolonged CPB, offering a more individualized prediction of vulnerability than conventional frailty indices.^27^

### Digitalization of Perfusion: from Monitoring to Prediction

Digital transformation is revolutionizing the heart–lung machine from a mechanical pump into an intelligent clinical platform. This analog model, while foundational, provides only static snapshots of a dynamic physiology. Parameters such as DO_2_, SvO_2_, and lactate levels are monitored at discrete intervals and integrated subjectively into perfusion decisions.^28^^,^^29^^,^^30^ The digitization of perfusion through continuous high-frequency data acquisition, machine learning-based risk modeling, and digital twin simulation enables unprecedented granularity in physiologic monitoring.^20^^,^^31^^,^^32^ These systems integrate real-time signals flow rate, pressure, hematocrit, DO_2_, temperature, and oxygen consumption into adaptive algorithms capable of forecasting hemodynamic instability or metabolic distress before clinical deterioration manifests. The concept of a digital twin a computational replica of the patient’s cardiovascular system extends this potential even further, allowing predictive simulation of perfusion adequacy under varying operative conditions. In this context, the concept of a digital twin warrants early clarification. A digital twin refers to a computational, continuously updated virtual representation of a patient’s physiological system, generated by integrating real-time intraoperative data with predictive models. In cardiopulmonary bypass, such a model aims to simulate individual hemodynamic and metabolic responses under varying perfusion conditions, thereby supporting personalized and anticipatory decision-making. Through these innovations, digital health integration could reframe CPB duration not as a static procedural constraint but as a dynamic, data-informed variable that can be optimized in real-time. Predictive analytics, coupled with automated or semi-automated perfusion control, may help identify the threshold beyond which prolonged bypass becomes harmful and allow surgeons and perfusionists to intervene pre-emptively. As cardiac surgery transitions from experience-based to data-driven precision, the convergence between surgical innovation and digital intelligence offers a unique opportunity to reconcile minimal invasiveness with physiological safety. By integrating robust data pipelines, predictive modeling, and interoperable digital ecosystems, the next generation of perfusion management could transform the way we understand and mitigate time-dependent surgical risk. Delays between sampling, analysis, and interpretation can obscure rapid hemodynamic fluctuations, leaving perfusionists to respond after physiological imbalance has already developed. In contrast, the digital era enables a proactive paradigm based on continuous online monitoring and real-time computation. Modern extracorporeal systems integrate sensors, high-resolution flow meters, and automated data acquisition modules capable of recording thousands of data points per minute (20-25). Continuous variables, such as DO_2_, carbon dioxide production (VCO_2_), venous saturation, and pressure-flow relationships, are streamed into integrated dashboards updated second by second. This shift from intermittent to continuous surveillance allows for trend-based reasoning the detection of perfusion deterioration before it becomes clinically apparent. However, digitalization alone is not sufficient. The key lies in data consistency across hardware, software, and institutional networks. Without harmonized data streams, even the most advanced monitoring systems remain underexploited. Once consistency is achieved, machine learning algorithms can extract predictive features from the complex interplay among flow, pressure, hematocrit, and metabolic parameters.^33^ Real-time models can identify hypoperfusion patterns and estimate the probability of acute kidney injury (AKI) by analyzing time–dose relationships between indexed oxygen delivery (DO_2_i), duration below critical thresholds, perfusion pressure, and metabolic demand. Rather than predicting AKI as a binary event, these models generate continuously updated risk probabilities, allowing early identification of patients exposed to harmful perfusion trajectories. Clinical studies have reported that prolonged exposure to low DO_2_i during CPB is independently associated with postoperative AKI, supporting the biological plausibility of such predictive frameworks. Importantly, these algorithms are intended as decision-support tools, complementing clinical judgment rather than replacing it.^23^^,^^33^ These scores, continuously recalculated during bypass, represent the first step toward algorithmic prediction.

Digital perfusion thus evolves through 3 distinct phases:

1. Monitoring: continuous acquisition of raw physiological signals;
2. Prediction: transformation of those signals into dynamic risk probabilities through machine learning models;
3. Decision-support: integration of predictive analytics into the perfusion workflow, providing automated alerts or adaptive control of pump flow, temperature, and gas exchange.

The culmination of this evolution is the digital twin, a computational replica of the patient’s cardiovascular and metabolic system. The digital twin continuously assimilates intraoperative data, simulates the outcome of potential interventions, and provides surgeons and perfusionists with personalized, evidence-based recommendations. In the context of prolonged CPB, this technology could redefine the meaning of time, transforming it from a passive measure of procedural duration into a controllable dimension of physiological performance (Table).^34^ To enhance clinical interpretability, each component of the proposed digital framework can be illustrated through existing and readily available use cases. For example, continuous monitoring of DO_2_i and VCO_2_ already enables real-time assessment of the balance between oxygen supply and metabolic demand. A progressive decline in DO_2_i relative to VCO_2_ during prolonged CPB may prompt early corrective actions, such as targeted increases in pump flow or perfusion pressure, before overt metabolic deterioration occurs. Similarly, machine learning-based models trained on high-frequency perfusion data have demonstrated the ability to estimate the probability of AKI intraoperatively, allowing perfusionists and surgeons to adapt strategies proactively rather than reactively. These examples highlight how the proposed framework builds upon technologies that are increasingly available in contemporary cardiac surgery practice.

**Table:** Evolution of CPB: From Conventional to Predictive Perfusion

Aspect	Traditional CPB System	Modern/Predictive CPB System
Monitoring	Intermittent manual recording (arterial pressure, venous saturation, temperature)	Continuous high-frequency multimodal monitoring (flow, DO_2_, VCO_2_, SvO_2_, lactate, hemodynamics)
Data acquisition	Isolated console readouts; limited interoperability	Integrated digital platforms connecting perfusion, anesthesia, and surgical systems
Data management	Paper-based or static digital logs	Automated data capture, cloud storage, and real-time dashboards
Decision-making	Operator-dependent, reactive adjustments	Algorithm-assisted, predictive, and adaptive control systems
Perfusion targets	Fixed flow/pressure settings	Personalized targets based on real-time metabolic demand and patient-specific modeling
Oxygen delivery (DO_2_)	Periodic manual sampling	Continuous online DO_2_ monitoring and indexed thresholds (DO_2_i)
Risk prediction	Post-hoc assessment based on outcomes	Real-time risk forecasting using machine learning algorithms
Integration level	Stand-alone heart–lung machine	Fully interoperable ecosystem with EHR, IoT sensors, and AI support
Human interaction	Manual control and interpretation	Semi-automated supervision with decision-support alerts
Conceptual model	Time and flow as fixed procedural parameters	Time and flow as dynamic, controllable physiological variables

Abbreviations: CPB, cardiopulmonary bypass; DO_2_, oxygen delivery; EHR, electronic health record; IoT, internet of things; SvO_2_, mixed venous oxygen saturation; VCO_2_, carbon dioxide production.

### From Data Consistency to Digital Intelligence

**Figure 2:**
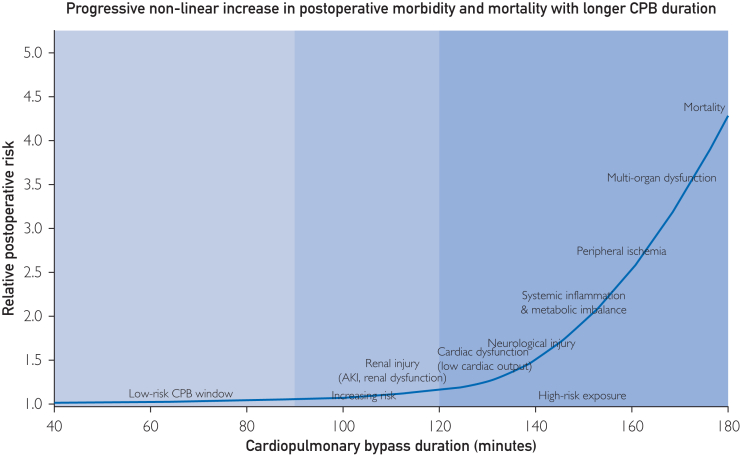
Rethinking cardiopulmonary bypass management in the digital health era. Digital integration transforms cardiopulmonary bypass from a static procedural parameter into a predictive, data-driven process. Continuous multimodal monitoring and artificial intelligence-assisted modeling enable adaptive perfusion tailored to the frail patient.

The reliability of predictive perfusion management depends fundamentally on data integrity. Historically, perfusion data were siloed across consoles, anesthesia monitors, and laboratory systems, each operating within its own proprietary domain. This fragmentation prevented holistic interpretation and algorithmic learning.^23^^,^^35^ The shift toward interoperable data ecosystems linking perfusion consoles with electronic health records and internet of things sensors marks the foundation of a digitally intelligent perfusion environment. Establishing structured, standardized perfusion datasets allows cross-institutional analysis and validation of predictive models. With harmonized ontologies, researchers can benchmark performance metrics and train algorithms on diverse populations. Moreover, cloud-based integration of perfusion data enables real-time multicenter learning loops, accelerating innovation. Ultimately, data consistency represents the bridge between raw monitoring and artificial intelligence (AI). Without consistent inputs, predictive models fail; with them, perfusion becomes a continuously learning system capable of evolving with every case (Figure 2).

### Future Directions and Ethical Implications

The incorporation of AI and digital twins into intraoperative care and CPB management introduces not only innovation but also ethical responsibility. Predictive models must remain transparent, explainable, and subject to clinical oversight. Data governance and cybersecurity frameworks are essential to ensure that patient privacy and algorithmic fairness are upheld. In the coming years, digital perfusion is poised to move from concept to clinical standard. By coupling minimally invasive cardiac surgery access with real-time digital feedback, cardiac surgeons and perfusionists will be empowered to anticipate and mitigate the risks rather than react to them. Continuous data consistency, validated predictive algorithms, and ethical AI integration will together transform the heart–lung machine from a support device into an intelligent physiological partner. Despite its potential, the implementation of digital perfusion and predictive analytics faces relevant logistical and organizational barriers. These include interoperability between perfusion platforms and hospital information systems, standardization of data formats, integration into established clinical workflows, and the need for adequate training of perfusionists and surgeons. Similar challenges have accompanied prior innovations in cardiac surgery, such as the adoption of minimally invasive approaches, transcatheter therapies, and enhanced recovery protocols, all of which required iterative refinement before widespread acceptance. Acknowledging these barriers is essential to ensure that digital perfusion frameworks remain clinically feasible and translational rather than purely theoretical.

## Conclusion

Minimally invasive and robotic cardiac surgery enhanced the evolution of modern cardiovascular care: less trauma, faster recovery, but paradoxically longer perfusion times. However, as evidence consistently shows, CPB and aortic cross-clamp are somehow inevitably associated with such minimal access, and those remain among the strongest predictors of postoperative morbidity and mortality, especially in frail patients with reduced physiological reserve. Traditional approaches have treated time as an unavoidable by-product of surgical complexity. However, the convergence of digital health, high-frequency data monitoring, and AI now enables a conceptual shift from passive observation to more timely and active control. Digital perfusion platforms integrate real-time physiologic signals into continuous risk models, allowing early detection of hypoperfusion, metabolic imbalance, and organ stress. Machine learning-based algorithms, coupled with digital twin simulations, can personalize flow and oxygen delivery, anticipating deterioration rather than reacting to it. These advances redefine the meaning of CPB duration: not merely how long, but how well this most likely prolonged time is managed. The future of cardiac surgery will depend on data consistency, interoperability, and ethical AI integration. Predictive perfusion analytics can reconcile minimal invasiveness with physiological precision, ensuring that digital intelligence enhances and not replaces clinical expertise. By embedding predictive technologies into the perfusion circuit, cardiac surgery can transition toward a new paradigm: while CPB time is unlikely to cease being a risk variable, its detrimental impact may be attenuated through improved physiological control, and the duration of low-risk bypass may be meaningfully prolonged through precision, data-driven perfusion management.

## Potential Competing Interests

The authors Mr Dsouki and Dr Condello are employees of Spectrum Medical Ltd. However, the present work was conducted independently as part of the authors' academic activity. Spectrum Medical did not provide any financial or material support and had no influence on the content of this manuscript. Dr Lorusso is consultant for Medtronic, Livanova (past) and J&J MedTech, member of the Xenios, Hemocue, ChinaBridge Medical and Eurosets (past) Medical Advisory Board.

## Declaration of Generative AI and AI-Assisted Technologies in the Writing Process

During the preparation of this work, the authors used ChatGPT-5 (OpenAI) in order to assist with English language correction and style refinement. After using this tool, the authors reviewed and edited the content as needed and takes full responsibility for the content of the publication.
